# An Interesting Case of Hemophagocytic Lymphohistiocytosis in a Postpartum Female With Recent COVID-19 Vaccination

**DOI:** 10.1155/carm/1876178

**Published:** 2024-11-25

**Authors:** John Patresan, Amardeep Kalsi, Perry Cook

**Affiliations:** ^1^Department of Medicine, New York Presbyterian Brooklyn Methodist, Weill Cornell Medicine, Brooklyn, New York, USA; ^2^Department of Hematology/Oncology, New York Presbyterian Brooklyn Methodist, Weill Cornell Medicine, Brooklyn, New York, USA

## Abstract

Hemophagocytic lymphohistiocytosis is a life-threatening cryptogenic inflammatory process. In some cases, the etiology is obscure, while in others multiple potential etiologies may be present. Diagnosis relies on observed findings rather than context.

## 1. Introduction

Hemophagocytic lymphohistiocytosis (HLH) is a rare entity characterized by an uncontrolled hyperinflammatory state which results in proliferation of histiocyte-associated phagocytosis of hematopoietic elements in the bone marrow along with massive cytokine release [1]. The major manifestations of HLH are fever (> 38 degrees Celsius), cytopenia affecting at least two lineages, hepatosplenomegaly, liver dysfunction, elevated levels of ferritin (> 500 ng/mL), hypertriglyceridemia (> 400), hypofibrinogenemia, low or absent natural killer cell activity, elevated soluble CD 25, and morphologic evidence of hemophagocytosis, usually on bone marrow examination. HLH-2004 diagnostic criteria require at least five out of the eight aforementioned criteria. HLH is categorized as primary where hereditary factors influence the syndrome and generally occurs at a very young age. There is also secondary HLH, which is classically associated with various pathologies, such as infection, malignancy, and autoimmune disease [2]. These origins are not mutually exclusive. Primary HLH can present at any age and underlying genetic mutations are not apparent in all primary HLH patients. Both types of HLH can be triggered by a variety of infectious or malignant processes. Here, we review a case of HLH potentially triggered by pregnancy which has been previously linked to HLH or possibly related to COVID messenger MRNA vaccination.

## 2. Case Report

A 22-year-old Hispanic female with gestational diabetes and hyperlipidemia who was status post spontaneous vaginal delivery of a healthy full-term child complicated by possible chorioamnionitis presented 5 days postpartum with 2 days of persistent fevers and increasing chills. Cefazolin was given but fever prompted a presentation to the emergency room. She was admitted to the gynecology service, febrile to 38.9 degrees Celsius, but hemodynamically stable. Her laboratory blood work at this time, demonstrated a bandemia with a mild anemia and transaminitis (Table 1). IV gentamicin/clindamycin/ampicillin was given, her blood and urine cultures were negative, and she was discharged after 2 days of intravenous antibiotics.

She was readmitted 6 days later with progressive fatigue, malaise, nausea, vomiting, fever, and chills. She complained of diffuse pain and headache. She had one episode of nonbilious nonbloody emesis. There was minor vaginal bleeding without abdominal or breast pain. She reported a temperature to 40.3 degrees Celsius. She received the second dose of COVID vaccine (Pfizer) 1 week prior to delivery. She traveled to Puerto Rico 6 months before delivery without other travel. No supplement use, homeopathic medicines, or any substance abuse history. Medical history notable for three prior spontaneous miscarriages including spontaneous abortion in 2015, an ectopic pregnancy in 2016, and a molar pregnancy necessitating dilation and curettage in 2018. She also had a remote history of 2 episodes of orbital cellulitis as a teenager responsive to systemic steroids. There was no family history of malignancy or autoimmune disease.

**Figure 1:**
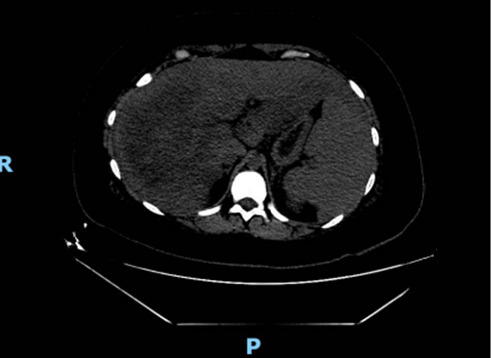
CT abdomen/pelvis with/without contrast demonstrating focal steatosis of the liver.

The readmission vital signs were 39° Celsius, 110 beats per minute, respiratory rate of 14 breaths per minute, blood pressure of 100/67 mmHg, saturation of 98% on room air. Physical exam notable for apparent acute distress, diaphoresis, tachycardia, and hepatosplenomegaly. There was no palpable lymphadenopathy. At this time, laboratory testing revealed no leukocytosis with a mild anemia which was known previously, worsening transaminitis with an elevated lactate dehydrogenase. Of note, her coagulation profile at this time was unremarkable (Table 2). The initial concern was possible urinary tract infection or endometritis, prompting empiric intravenous piperacillin-tazobactam. Infectious disease and gastroenterology were consulted with concern of autoimmune or drug-induced liver injury versus acute fatty liver of pregnancy. Further work up including autoimmune and viral serologies was ultimately negative (Table 2). CT of abdomen/pelvis revealed focal steatosis of the liver without evidence of any uterine/pelvic infection (Figure 1). MRCP did not show biliary obstruction.

Given an absent explanation of the liver dysfunction, liver biopsy on hospital day 6 was obtained, which suggested drug-induced liver injury (mild macrovesicular, small droplet, and focal microvesicular steatosis, approximately 10% with portal and lobular inflammatory activity containing lymphocytes, eosinophils, and plasma cells).

Comprehensive infectious work up including urine studies, blood cultures, gonorrhea and chlamydia testing, parasite testing, aforementioned viral serology and COVID-19 PCR were negative. Her empiric antibiotics were stopped. On hospital day 7, rheumatology was consulted for persistent fevers and overall lack of clinical improvement. HLH/macrophage activation syndrome was considered and repeat labs revealed bicytopenia now with worsening anemia, hypofibrinogenemia, an elevated D-Dimer, hyperferritinemia, increased triglycerides (Table 3). Her coagulation profile at this juncture remained within normal limits.

**Figure 2:**
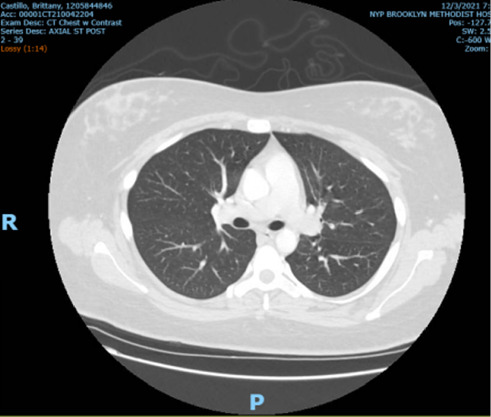
CT chest with contrast which did not demonstrate and remarkable findings.

Hematology was now consulted for the question of HLH. Peripheral blood film was unremarkable except for decreased WBC with left shift. A CT chest with contrast was performed at this time to evaluate for any malignancy which was unremarkable (Figure 2).

**Figure 3:**
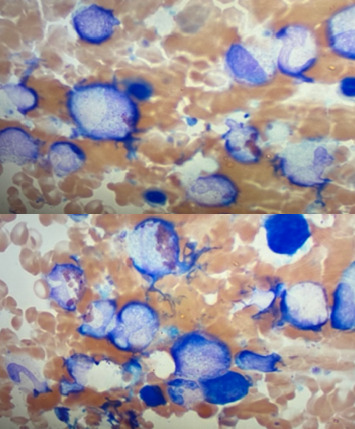
Bone marrow biopsy demonstrating hemophagocytosis of both erythrocytic and neutrophilic elements.

It was decided to pursue a bone marrow biopsy to help cinch the diagnosis on hospital day 8. Bone marrow biopsy revealed a cellular marrow (∼80%) with prominent hemophagocytosis of both erythrocytic and neutrophilic elements (Figure 3).

Biopsy findings prompted methylprednisolone 1000 mg IV every 24 h for 3 days. Soluble IL-2 receptor was measured at 37,134 pg/mL or approximately 4000 U/mL (325–1785 pg/mL or 241–846 U/mL) (Table 4). Next-generation sequencing to detect genetic alterations seen in hereditary HLH was also obtained, which was ultimately negative (Table 4). After her 3 days of pulse methylprednisolone, she was then transitioned to oral prednisone (1 mg/kg day). Her fevers eventually remitted, and pain resolved. Etoposide/tacrolimus were considered if clinical deterioration persisted; however, she responded dramatically with normalization of liver function tests, lactate dehydrogenase, ferritin, D-Dimer, and resolution of leukopenia which allowed discharge with close outpatient follow up. She was tapered from 80 mg daily of prednisone slowly over 5 months and was followed for an additional 3 months for laboratory monitoring afterwards. She continued to remain asymptomatic without need for re-challenging steroids or escalating care with immunosuppressants/cytotoxic chemotherapy.

## 3. Discussion

This case demonstrates the diagnostic challenge of HLH in a young postpartum female with a recent COVID vaccination first suspected of having an infectious cause of fevers. The patient ultimately met seven out of the eight 2004 HLH criteria including fever, cytopenia of at least two cell lines, splenomegaly, elevated ferritin, hypertriglyceridemia, hypofibrinogenemia, elevated soluble IL-2 receptor levels, and evidence of hemophagocytosis on bone marrow evaluation. It should also be noted that sIL-2r > 2400 U/mL is very sensitive for adult HLH, sIL-2r < 2400 is helpful for ruling out HLH, and > 10,000 U/mL is helpful for ruling it in [3].

Our patient's H score (described by Fardet et al.) was 309 which suggests a > 99% probability of hemophagocytic syndrome. The clinical presentation stems from excessive release of proinflammatory cytokines including IL-6, tumor necrosis factor-alpha, and interferon-gamma which can cause multiorgan failure involving the bone marrow, liver, spleen, and central nervous system [4]. The patient's work-up did not elucidate any obvious infection or evidence of lymphoma, other hematologic malignancy, or rheumatologic disease. No genetic predisposition was identified. Thus, pregnancy and/or COVID vaccination remain as potential precipitating causes in this case. The diagnosis of HLH in pregnancy can be challenging as there are other conditions that can present similarly such as HELLP and acute fatty liver of pregnancy [5]. The pathophysiology of pregnancy-induced HLH is not understood but is increasingly recognized. It is postulated that maternal T-helper lymphocytes typically shift from Th1 predominance to Th2 to adapt to the growing fetus, resulting in increased susceptibility to viral infections in the context of decreased cell-mediated immunity [6]. Perhaps the decreased cell-mediated immunity facilitates hemophagocytosis as the primary immune response for infection instead of the Th1 immune response [7]. Another purported mechanism is that the placenta during gestation releases syncytiotrophoblast components, which are fetal-derived DNA and RNA components that enter the maternal circulation and cause alloimmunization, hence a systemic inflammatory response such as HLH [8].

Familial or primary HLH is extremely rare in the adult population and almost occurs exclusively in the pediatric population, typically in the first 2 years of life [9]. Although it was a possibility that should be explored despite its rarity, it was ruled out in our patient case. The immunologic mechanism between the primary and secondary HLH is important to understand. It is crucial to recognize that malignant neoplasms are frequently associated with HLH in both children and adults. HLH can often mask the diagnosis of malignancy; however, can also sometimes present as a sequela of malignancy, such as in intravascular B cell lymphoma, where it is appreciated in the Asian variants [10]. Most neoplasms associated with HLH are hematologic malignancies, although solid tumors including hepatocellular carcinoma, lung, and prostate have been noted in the literature [10]. Among the hematologic malignancies, NK/T-cell lymphomas and acute lymphocytic leukemias, Hodgkin's lymphoma and multiple myeloma are often described; HLH is rarely discovered in patients with non-Hodgkin B-cell lymphomas [10]. The proposed mechanism is not clearly understood, although it is postulated that the pathogenesis could be driven by impairment of the cytotoxic pathway by the neoplasms through neoplastic changes in the cytotoxic cell itself or through malignancy-associated immune dysregulation [10].

Another syndrome which should be evaluated for in this setting would be multisystem inflammatory syndrome in adults (MIS-A). There have been reports in the literature of MIS-A ensuing after both SARS-CoV-2 infection and the COVID-19 vaccination as well [11]. MIS-A is a febrile hyperinflammatory syndrome which can present with fever of 38.0 Celsius > 24 h with clinical features of end-organ damage including severe cardiac illness, new-onset neurologic signs, shock or hypotension, abdominal symptoms, thrombocytopenia, elevated serum inflammatory markers and laboratory evidence of a positive SARS-CoV-2 infection [11]. Our patient did not quite fit these criteria, as she had no evidence of cardiac dysfunction, encephalopathy or shock. She had overwhelming evidence to have secondary HLH instead as she met seven out of the eight HLH criteria with sIL-2r > 2400 U/mL which is something you would not see in MIS-A. She was also pancytopenic with bone marrow morphologic evidence of hemophagocytosis which is another feature you would not expect in MIS-A. However, it is essential for the clinician to at least consider MIS-A in the differential diagnosis of a hyperinflammatory state after a patient receives a COVID-19 vaccination.

An additional question is whether the COVID vaccine (administered 1 week prior to delivery) could have been the inciting event. There was a case in the literature of a 36 year old healthy female who reportedly developed secondary HLH from the COVID vaccine (Oxford Astra-Zeneca) [11]. Although COVID vaccinations have been reported to be related to certain immune phenomena such as ITP and vaccine-induced thrombotic thrombocytopenia (VITT), HLH is a rare consequence of vaccinations, infrequently reported after influenza vaccines [11]. Various other vaccines including tetanus, pneumococcal, diphtheria-pertussis-tetanus, and mumps-rubella-measles vaccine have also been implicated in triggering secondary HLH, suggesting a possible underlying mechanism of an exaggerated immune response [12]. Regarding the COVID BNT162b2 formulation, some literature has postulated that the hyperinflammatory state could be brought on by the inflammatory generating potential of spike proteins, the adaptive and innate immune mechanisms including molecular mimicry and the potential immune response mediated by the antispike antibodies themselves [13].

One article also postulates that excessive interleukin-6 (IL-6) amplification can cause a cytokine storm after COVID-19 vaccination [14]. IL-6 can then induce a signal transduction pathway via STAT-3 and NF-kB (via IL-6 activation of tumor necrosis factor-*α*) which draws immunocompetent cells through the action of chemokines. These cells then propagate the vicious cycle of IL-6 amplification, and an hyperinflammatory state ensues [14]. Although the direct causality of vaccine-induced HLH has yet to be elucidated, heightened monitoring and reporting of such cases are pivotal in the future to help gather data and ascertain the relationship between the two. As of March 2022, there were approximately 50 cases of secondary HLH from various formulations of the COVID vaccine reported in the vaccine adverse effects reporting system and this number will certainly continue to increase as time goes on [14]. It is also important to note that before jumping to the conclusion of a COVID vaccine induced secondary HLH, a background infection such as an active Epstein–Barr or other common virus should be excluded. There have been cases where there exists HLH induced by COVID-19 vaccination on a chronic EBV infection background [15].

It is prudent to recognize whether the SARS-CoV-2 infection itself could be a potential driver of HLH. There have been several case reports in the literature suggesting this possibility. The proposed mechanism of COVID-19-triggered secondary HLH is thought to be due to dysregulation of the immune response and an inflammatory reaction to the virus. This can potentially occur in a wide range of patients with PCR-proven recovery [16].

The challenge lies with diagnosing HLH in the setting of SARS-CoV-2 infection, particularly in a septic patient, given the degree of overlap in the clinical features and laboratory presentation of secondary HLH and COVID-19, based on the H-Score. Therefore, a different set of criteria which incorporates variables from the H-score can be implemented to diagnose secondary HLH in this context [17]. In our case, however, the patient had negative SARS-CoV-2 nucleic amplification test making this less likely. Lastly, our case is somewhat nebulous as there were other confounding factors, such as the patient's pregnancy and delivery which coincided with her administration of the COVID-19 vaccination.

In conclusion, heightened attention and a broader scope should be employed by practitioners when encountering a patient with persistent pyrexia, organomegaly, etc. during pregnancy or in the immediate postpartum period as prompt diagnosis and therapy is pivotal for a favorable clinical outcome. In addition to the many etiologies of secondary HLH including various infections and malignancies; perhaps consideration of COVID vaccine-induced HLH can be added to the investigation in future scenarios where the trigger of HLH remains unclear. Lastly, providers should approach secondary HLH with a broad scope of iatrogenic triggers in addition to the commonly described provokers of HLH. The temporal relationship of the COVID vaccine in our case may be too remote to be contributory, although it is difficult to say there was no immunomodulatory effect given; we are in the primacy of its synthesis and delivery. Whether the COVID booster should be administered in such a case, must be weighed carefully, of course to avoid triggering another hyperinflammatory state. For future cases in which a patient is well-established to have secondary HLH, a careful evaluation of the risks and benefits of an additional COVID-19 booster should be undertaken in order to avoid a flare-up, so to speak.

Whether the patient wants to pursue another pregnancy requires careful shared decision-making between the provider and patient with meticulous monitoring throughout all trimesters. According to HLH-94, the standard of care included 8 weeks of initial chemotherapy (typically epipodophyllotoxins) in combination with corticosteroids in effort to achieve complete remission, followed by continuation therapy until an acceptable bone marrow donor could be found [18]. Fortunately, our patient briskly responded to high-dose steroid administration with subsequent slow taper over 8 weeks with complete resolution of symptoms, as well as improvement in her cytopenias and serum inflammatory markers. If she had evidence of refractory disease at that point of completion, cytotoxic chemotherapy would have been entertained with the possibility of allogenic stem-cell transplant. Of course, any chemotherapy has its potential risks, such as secondary acute myeloid leukemia later in life which has been described with the use of topoisomerase-II inhibitors [19]. This patient was quite young; therefore, the institution of chemotherapy should be carefully evaluated and utilized if truly necessary. If the COVID vaccine is identified as a driver of HLH, whether the conventional etoposide and steroid-based treatment protocol should be the standard of care, is another question that should be answered.

## Figures and Tables

**Table 1:** Labs on initial presentation.

Laboratory test (reference range)	Result
WBC (4.5–11.0 × 10^3^ *μ*L, bands < 10%)	8.83 × 10^3^ *μ*L, 29% bands
Hemoglobin (female: 12.1–15.1 g/dL)	11.0 g/dL
Platelets (150–450 × 10^3^ *μ*L)	298 × 10^3^ *μ*L
Lactate (0.5–2.2 mmol/L)	1.12 mmol/L
AST (8–33 IU/L)	277 IU/L
ALT (4–36 IU/L)	444 IU/L
Alkaline phosphatase (44–147 IU/L)	216 IU
Total bilirubin (0.1–1.2 mg/dL)	1.3 mg/dL
Direct bilirubin (< 0.3 mg/dL)	0.49 mg/dL

**Table 2:** Readmission labs.

Laboratory test (reference range)	Result
WBC (4.5–11.0 × 10^3^ *μ*L, bands < 10%)	5.15 × 10^3^ *μ*L, 9% bands
Hemoglobin (female: 12.1–15.1 g/dL)	10.5 g/dL
Platelets (150–450 × 10^3^ *μ*L)	216,000 *μ*L
AST (8–33 IU/L)	618 IU/L
ALT (4–36 IU/L)	883 IU/L
Alkaline phosphatase (44–147 IU/L)	338 IU
Lactate dehydrogenase (105–333 IU/L)	1673 IU/L
INR/PT (0.8–0.1/11.0–13.5)	1.01/11.7
aPTT (25–35 s)	27.8
Total bilirubin (0.2–1.3 mg/dL)	1.1
ESR (0–29 mm/hr)	49 mm/hr
CRP (< 10 mg/L)	65 mg/L
Antinuclear antibody ANA	Negative
Antimitochondrial antibody	Negative
Antismooth muscle antibody	Negative
EBV (IgM and IgG)	Negative
CMV (IgM and IgG)	Negative
HSV (IgM)	Negative
Hepatitis A, B, C, E (IgM and IgG)	Negative
HIV ab	Negative

**Table 3:** Readmission hospital day 7 labs.

Laboratory test (reference range)	Result
WBC (4.5–11.0 × 10^3^ *μ*L, bands < 10%)	2.32 × 10^3^ *μ*L, 24% bands
Hemoglobin (female: 12.1–15.1 g/dL)	9.3 g/dL
Fibrinogen (200–400 mg/dL)	139 mg/dL
D-Dimer (220–500 ng/mL)	16,754 ng/mL
LDH (105–333 IU/L)	2087 units/L
Ferritin (24–336 *μ*g/L)	23,755 *μ*g/L
Triglycerides (< 150 mg/dL)	417 mg/dL
INR/PT (0.8–0.1/11.0–13.5)	1.15/13.4
aPTT (25–35 s)	28.7

**Table 4:** Readmission hospital day 7 labs.

Laboratory test (reference range)	Result
Soluble IL-2 receptor (325–1785 pg/mL or 241–846 U/mL)	37,134 pg/mL or approximately 4000 U/mL
PRF1	Negative
RAB27	Negative
XLP	Negative
MUNC 13-4	Negative
STXBP2	Negative
STX11	Negative

## Data Availability

The data supporting the findings of this case report are available from the corresponding author upon reasonable request.

## References

[1] George M. R. (2014). Hemophagocytic Lymphohistiocytosis: Review of Etiologies and Management. *Journal of Blood Medicine*.

[2] Zahir H., Belkhir J., Mouhib H., Ait Ameur M., Chakour M. (2019). Hemophagocytic Lymphohistiocytosis: Epidemiological, Clinical and Biological Profile. *Turkish Journal of Medical Sciences*.

[3] Hayden A., Lin M., Park S. (2017). Soluble Interleukin-2 Receptor is a Sensitive Diagnostic Test in Adult HLH. *Blood advances*.

[4] Keenan C., Nichols K. E., Albeituni S. (2021). Use of the JAK Inhibitor Ruxolitinib in the Treatment of Hemophagocytic Lymphohistiocytosis. *Frontiers in Immunology*.

[5] He M., Jia J., Zhang J. (2017). Pregnancy-Associated Hemophagocytic Lymphohistiocytosis Secondary to NK/T Cells Lymphoma: A Case Report and Literature Review. *Medicine*.

[6] Wang L. Y., Hu J., Ramsingh G. (2018). A Case of Recurrent Pregnancy-Induced Adult Onset Familial Hemophagocytic Lymphohistiocytosis. *World Journal of Oncology*.

[7] Tumian N. R., Wong C. L. (2015). Pregnancy-Related Hemophagocytic Lymphohistiocytosis Associated With Cytomegalovirus Infection: A Diagnostic and Therapeutic Challenge. *Taiwanese Journal of Obstetrics & Gynecology*.

[8] Liu L., Cui Y., Zhou Q., Zhao H., Li X. (2021). Hemophagocytic Lymphohistiocytosis during Pregnancy: A Review of the Literature in Epidemiology, Pathogenesis, Diagnosis and Treatment. *Orphanet Journal of Rare Diseases*.

[9] Memon F., Ahmed J., Malik F., Ahmad J., Memon D. (2020). Adult-Onset Primary Hemophagocytic Lymphohistiocytosis: Reporting a Rare Case With Review of Literature. *Cureus*.

[10] Verma A., Sharma A., Robetorye R., Porter A., Hilal T. (2020). Intravascular Large B-Cell Lymphoma Associated With Systemic and Central Nervous System Hemophagocytic Lymphohistiocytosis: A Case Report. *The Permanente Journal*.

[11] Rosado F. G. N., Kim A. S., Kim A. S. (2013). Hemophagocytic Lymphohistiocytosis: An Update on Diagnosis and Pathogenesis. *American Journal of Clinical Pathology*.

[12] Narvel H., Kaur A., Seo J., Kumar A. (2022). Multisystem Inflammatory Syndrome in Adults or Hemophagocytic Lymphohistiocytosis: A Clinical Conundrum in Fully Vaccinated Adults With Breakthrough COVID-19 Infections. *Cureus*.

[13] Cory P., Lawrence H., Abdulrahim H., Mahmood-Rao H., Hussein A., Gane J. (2021). Lessons of the Month 3: Haemophagocytic Lymphohistiocytosis Following COVID-19 Vaccination (ChAdOx1 nCoV-19). *Clinical Medicine*.

[14] Nasir S., Khan S. R., Iqbal R., Hashmi A. P., Moosajee M., Nasir N. (2022). Inactivated COVID-19 Vaccine Triggering Hemophagocytic Lymphohistiocytosis in an Immunocompetent Adult-A Case Report. *Journal of Clinical and Translational Research*.

[15] Iwamura N., Eguchi K., Koga T. (2023). Hypocomplementemic Urticarial Vasculitis Case With Hemophagocytic Lymphohistiocytosis Following SARS-CoV-2 mRNA Vaccination. *Immunological Medicine*.

[16] Lin T. Y., Yeh Y. H., Chen L. W. (2022). Hemophagocytic Lymphohistiocytosis Following BNT162b2 mRNA COVID-19 Vaccination. *Vaccines*.

[17] Wu V., Lopez C. A., Hines A. M., Barrientos J. C. (2022). Haemophagocytic Lymphohistiocytosis Following COVID-19 mRNA Vaccination. *BMJ Case Reports*.

[18] Jordan M., Allen C., Weitzman S., Filipovich A., McClain K. (2011). How I Treat Hemophagocytic Lymphohistiocytosis. *Blood*.

[19] Bellomo J., Cabrol C., Beris P., Mermillod B., Zulian G. B., Alberto P. (1993). Etoposide and Secondary Haematological Malignancies: Coincidence or Causality?. *Annals of Oncology*.

